# Editorial: Advances in plant cultivation and physiology of oilseed crops

**DOI:** 10.3389/fpls.2023.1280392

**Published:** 2023-09-18

**Authors:** Hui Zhang, Guangsheng Zhou, Rajeev K. Varshney, Shubo Wan

**Affiliations:** ^1^Department of Crop Science and Technology, College of Agriculture, South China Agricultural University, Guangzhou, China; ^2^College of Plant Science and Technology, Huazhong Agricultural University, Wuhan, China; ^3^State Agricultural Biotechnology Centre, Centre of Crop and Food Innovation, Agriculture & Food Security with Food Futures Institute, Murdoch University, Perth, WA, Australia; ^4^Institute of Crop Germplasm Resources, Shandong Academy of Agricultural Sciences, Jinan, China

**Keywords:** oilseed crops, cultivation, microecology, rhizobium, candidate gene

## Introduction

Primarily cultivated for the oil present in their seeds, major oilseed crops include rapeseed (*Brassica napus* L.), soybeans (*Glycine max*), and peanuts (*Arachis hypogaea* L.). Given the growing global demand for vegetable oils and the high agronomic value of these plants, oilseed crops have been planted at increasingly high levels throughout the world. When growing oilseed crops, factors that need to be taken into consideration include oil composition, quality, and yield, all of which can be influenced by genetic factors, environmental factors, and interactions between genetics and the environment. In order to maximize oil output, it is essential that an appropriate cultivation environment be maintained. Despite the importance of oilseed crops to societies throughout the world, advances in the cultivation of these crops have lagged behind those for major grain crops. Most oilseed crops exhibit root nodules that facilitate nitrogen fixation mediated through symbiotic *Rhizobium* interactions. Succession cropping-related challenges are one of the primary obstacles to the optimization of oilseed planting efforts. As such, there remains a pressing need to develop appropriate techniques for the robust and reliable cultivation of oilseed crops.

Research related to efforts to improve oilseed crop performance can be classified broadly into studies of Crop and Product Physiology and studies of Plant Breeding, covering a broad range of topics related to the mechanisms governing oilseed crop growth and optimal cultivation strategies. These analyses include physiological and molecular efforts to determine the factors associated with high-quality oil crop yields, studies of oilseed plant biotic and abiotic stress resistance research mechanisms, analyses focused on the efficiency of resource utilization by these crops (particularly with respect to nitrate and calcium metabolism), efforts to overcome obstacles to the succession cropping of oilseed plants or to improve yields through intercropping, and research focused on the microecological characteristics and regulation of these oilseed crops. The goal of these efforts is to facilitate sustainable oilseed crop production by enhancing the efficiency of water and fertilizer use, reducing fertilizer and pesticide inputs, narrowing yield gaps, improving oil quality, and applying cutting-edge research findings to the field as a means of improving cultivation efforts. In this Research Topic, the contributed studies primarily focus on oilseed crops, although they are not restricted to studies of soybeans, peanuts, and rapeseed.

Three studies have been accepted and included that are related to Crop and Product Physiology. Li et al. performed experiments focused on cross- and self-pollination at 1 d or 3-4 d before flowering as a means of exploring the molecular basis for late-acting self-incompatibility (LSI) in *Camellia oleifera*. They employed WGCNA and RNA-Seq approaches to identify LSI-related differentially expressed genes (DEGs), with subsequent GO and KEGG enrichment analyses revealing that the majority of these DEGs were associated with the plant hormone signal transduction, ubiquitin-mediated proteolysis, ABC transporters, brassinosteroid biosynthesis, flavonoid biosynthesis, and the stilbenoid, diarylheptanoid and gingerol biosynthesis pathway. A subset of these DEGs was validated via qRT-PCR, providing a foundation for future efforts to improve *C. oleifera* yields. In a second study conducted by Wu et al., the researchers analyzed the relationship between nitrogen (N) fertilization and rhizosphere soil carbon emissions in a continuous peanut monoculture system with a focus on changes in the abundance of cellulose-specific microbes. They found that the effects of N fertilization on the rhizosphere bacterial and fungal communities varied over the 5-year monoculture period as a function of N levels. The application of N at 120 kg hm^−2^ (N120) was associated with the proliferation of both alphaproteobacteria and gammaproteobacteria, with the levels of these bacteria being positively correlated with root biomass and the activity of b-D-cellobiohydrolase. Soil respiration rates were also increased under N120 fertilization conditions. With changes in cellulose-related microbial communities and the activity of cellulose decomposition-related enzymes, N fertilization was ultimately found to impact C dynamics in the rhizosphere soil. By linking microbial communities and soil C cycling, this study offers mechanistic insight into how rhizosphere soil microbial communities can respond to N input under continuous monocropping conditions. These results represent an important reference for efforts to preserve the long-term viability of oilseed crops under intensive cultivation efforts while also better enabling the estimation of soil C emissions in the context of global climate change. In the third study, Zhou et al. utilized a controlled deterioration treatment (CDT) experimental paradigm to assess the process of safflower seed aging, which represents an important topic in the context of agricultural production. In order to better clarify the biological storage properties of seeds and to optimize seed life spans, research is necessary to define the critical node (CN) where safflower population viability decreases rapidly. Through their CDT-based artificial aging experiments, the authors were able to establish seed survival curves. These results, together with other analyses, led to the conclusion that the C10 timepoint is a CN during the aging of safflower seeds and that the CN was around a germination level of 86%.

One study has also been accepted and included in the Plant Breeding section. In this report, Wang et al. utilized RNA-Seq and GWAS approaches to identify 115 significant loci and 1066 DEGs related to the branch angle and dispersion degree in rapeseed plants (*Brassica napus* L.). These DEGs were primarily associated with auxin biosynthesis and transport, glycan degradation, indole alkaloid biosynthesis, cell extension, and fatty acid elongation. Their results provide a basis for future efforts focused on the genetic improvement of the branch architecture of rapeseed plants through molecular breeding.

We wish to express our gratitude to all the authors who participated in this project, contributing their relevant research and expertise to this effort. We hope that this Research Topic will serve as a key step forward toward the sustainable production of oilseed crops, offering a foundation that will stimulate additional beneficial research in this field.

## Author contributions

HZ: Investigation, Project administration, Validation, Writing – original draft, Writing – review & editing. GZ: Conceptualization, Project administration, Writing – review & editing. RV: Investigation, Supervision, Visualization, Writing – review & editing. SW: Conceptualization, Investigation, Project administration, Resources, Supervision, Validation, Visualization, Writing – review & editing.

